# Current pattern of ocular trauma as seen in tertiary institutions in south-eastern Nigeria

**DOI:** 10.1186/s12886-021-02162-4

**Published:** 2021-12-05

**Authors:** Chinwe Cynthia Jac-Okereke, Chukwunonso Azubuike Jac-Okereke, Ifeoma Regina Ezegwui, Rich Enujioke Umeh

**Affiliations:** 1 University of Nigeria Teaching Hospital, Ituku/Ozalla, Enugu State Nigeria; 2 Enugu State University of Science and Technology Teaching Hospital, Park Lane, GRA, Enugu State Nigeria

## Abstract

**Background:**

Ocular trauma is a leading cause of monocular blindness worldwide and in developing countries, are not only more common but also more severe. Industrialization and urbanization may alter or modify prevalent aetiological factors and the presentation of ocular trauma.

**Objective:**

To determine the current pattern of eye injuries in Teaching Hospitals in Enugu State.

**Methods:**

This prospective cross-sectional descriptive study was carried out at the eye clinics and emergency units of two tertiary institutions in Enugu, Nigeria. Consecutive ocular trauma patients were enrolled over a 5-month period. On presentation, they underwent ocular examination including visual acuity (which was categorized according to the WHO classification of visual impairment and blindness}, anterior and posterior segment examinations. Injuries were grouped using the Birmingham Eye Trauma Terminology system.

**Results:**

Within the study period, 2545 new patients presented to the hospitals where the study was conducted. A total of 89 patients presented with ocular trauma, giving an incidence of 3.5%. The male to female ratio was 1.3:1 and patients aged 10 to 19 years formed the majority (24%). Domestic accidents (22%) were the most common cause of ocular injuries while blunt objects (57%) were the most common agents. Closed globe injuries constituted 76% of all injuries. Forty-three per cent of patients presented within 24 h of injury.

**Conclusion:**

Ocular trauma is still prevalent in South-eastern Nigeria and there has indeed been a change in the pattern as well as the incidence. Assault and road traffic accidents have emerged as important causes of eye injuries. In addition, there has been an improvement in the health-seeking behaviour of people living in Enugu and this may reflect positively on the uptake of recommended preventive strategies.

## Background

Ocular trauma is an important cause of visual impairment and a leading cause of preventable monocular blindness [1]. Worldwide, there are approximately 1.6 million people blind from eye injuries, an additional 2.3 million people with bilateral low vision from this cause and almost 19 million with unilateral blindness or low vision [2].

In developing countries, eye injuries are not only more common [2] but also more severe in their effect and this may be attributed to socioeconomic background, inadequate safety measures, lack of optimum treatment facilities, use of traditional eye medication and poor education [2]. Studies from Nigeria and other parts of Africa have reported ocular trauma as an important cause of monocular blindness [3–6].

The spectrum of ocular injuries ranges from the very mild non-sight threatening to extremely serious with potentially blinding consequences. Based on the more comprehensive Birmingham Eye Trauma Terminology system (BETTS) [7–9], recent studies [10–12] have classified open-globe injury types as rupture, penetrating, intraocular foreign body, perforating and mixed; closed-globe injury types, as contusion, lamellar laceration, superficial foreign body and mixed. The rationale for classifying ocular trauma is to determine and document severity of injury. It also provides a standardized description and terminology for the injury, which is internationally accepted and understood. The type and extent of damage sustained by a traumatised eye depends on both the mechanism and force of the trauma [13]. Common consequences of ocular blunt trauma include periocular lid ecchymosis/ haematoma, orbital fractures, subconjunctival haemorrhage, corneal abrasions/ulcers, hyphaema, cataracts, lens dislocation/ subluxation, contusions, retinal detachments and globe rupture. Penetrating/ perforating injury could lead to lacerations of the eyelids, cornea or sclera which may be associated with intraocular haemorrhage, retained foreign bodies or tractional retinal detachment [13, 14].

In Nigeria, the causes of ocular trauma comprise a peculiar mix; ranging from domestic injuries, rural occupational hazards (farming, hunting), industrialized work-related trauma, road traffic accident-related and assault-related [15–18]. Available literature, in Nigeria, has also established a relationship between; domestic related trauma and gender, bilateral involvement and psychological status, and to determine commonly affected ocular structures [15, 19, 20]. Regarding domestic trauma, women and children are at greater risk due to their increased involvement with domestic activities [15]. While bilateral ocular trauma with blinding consequences is rare, reported cases have been associated with blast injuries, explosions and severe depression; chemical eye injuries have been documented as the most common cause [19]. The cornea, eye lids and conjunctiva have been reported as the most affected ocular structures in cases of traumatic eye injuries [20–22]. Most Nigerian studies on ocular trauma have been retrospective with a keen focus on prevalence as opposed to incidence and a bias for the paediatric population [10, 12, 23]. Recent prospective studies have however been carried out in South western Nigeria [21, 24].

Previous research in the index study area highlighted the incidence, trend and aetiological factors of ocular trauma in the nineteen-eighties [22]. Enugu State, South-Eastern Nigeria, has over the years become more industrialized with many manufacturing /processing industries, telecommunication firms and increasing professional jobs and these may have altered and/or modified aetiological risk factors of ocular trauma. Improved expressways and hence, increased vehicular movement**,** has been known to increase the incidence of road traffic accidents in urban communities Furthermore, classification of types and grades of ocular trauma has changed.

This study therefore explores the current pattern of ocular trauma in Enugu State, South-eastern Nigeria, against this background of possible changing aetiology in order to make new recommendations on improving eye health.

## Methods

This prospective cross-sectional descriptive study was carried out at the eye clinics and emergency units of the University of Nigeria Teaching Hospital (UNTH), Ituku/ Ozalla, Enugu and the Enugu State University of Science and Technology Teaching Hospital (ESUT TH), Park Lane, Enugu, both in Enugu state, Nigeria. All consecutive patients with ocular trauma presenting to the eye clinics or emergency units of the above hospitals,over a 5 month period (from 1st June to 31st October 2017), were enrolled.

Questionnaire administration, participant recruitment, patient examination and recording of findings was carried out by the authors, assisted by 3 ophthalmology residents from each centre who had received appropriate training during the pilot study.

Consecutive patients with ocular trauma were recruited from the clinics and accident and emergency units of both centres. A semi-structured questionnaire was administered by face-to-face interview to acquire socio-demographic information. Relevant history regarding the injury such as complaints, time interval before presentation, prior intervention, activity at time of injury and source of injury was also obtained. The information was obtained from a guardian/ parent for young children or an eye witness, where the patient was unable to supply it.

Patients were then examined and findings recorded. Visual acuity was assessed first, using an alphabet chart for literate adults or the tumbling E chart for illiterate adults and older children. Pre-school children had their visual acuity assessed using the HOTV charts and for younger children, age-appropriate methods of vision assessment were used. The visual acuities were categorized according to the World Health Organization (WHO) classification of visual impairment and blindness [25]. Examination of the anterior segment was carried out with a pen torch and a Slit lamp bio microscope. The posterior segment of the eye was examined by direct and indirect ophthalmoscopy, as indicated, after dilation of the pupils with Tropicamide 1% or Tropicamide/Phenylephrine 0.8%/5% eye drops.

The ocular injuries were categorized according to the Birmingham Eye Trauma Terminology System (BETTS) [26] into open and closed globe injuries.

The data were analysed using the IBM SPSS Statistics for Windows (version 23.0; IBM Corp., Armonk, NY). Frequencies, means and standard deviations were generated to observe patterns of variable distribution. *P* values < 0.05 were considered significant.

## Results

During the study period, 89 cases of ocular trauma presented to the study centres; 32 at UNTH and 57 at ESUT TH. The total number of new eye cases seen within the same period was 2545. This gave an incidence of ocular trauma of 3.5%. Eight of these patients were excluded from the study because consent was not given, either directly or by proxy. Therefore, a total of 81 patients were enrolled in the study.

The gender distribution of the patients was 45 (56%) male and 36 (44%) female with a male to female ratio 1.3:1. The ages of patients ranged from 6 months to 81 years, with an overall mean age of 28.4 ± 18.1 years (males 25.3 ± 15.8 and females 32.3 ± 20.3 years). The age group with the highest frequency was 10–19 years (24%) and over 75% of the patients were under the age of forty.

Most of the patients were single (68%), students (44%), and had a minimum of a secondary education (64%). The demographics of the patients are represented in Table 1.

**Table 1:** Demographics of Patients

	Number	Percentage
Gender		
Male	45	56%
Female	36	44%
Age		
0–9 years	12	15%
10–19 years	19	24%
20–29 years	18	22%
30–39 years	12	15%
40–49 years	6	7%
50–59 years	9	11%
≥60 years	5	6%
HEQ^a^		
No formal education	5	6%
Primary	24	30%
Secondary	37	46%
Tertiary	13	16%
Postgraduate	2	2%
Occupation		
Artisan	4	5%
Civil/ Public Servant	11	14%
Driver	3	4%
Entrepreneur	14	17%
Farmer	6	7%
Student	36	44%
Unemployed	7	9%

^a^*HEQ* Highest Educational Qualification

The laterality of the eyes affected was as follows; right eye (RE) 39 (48.1%), left eye (LE) 36 (44.4%) and both eyes 6 (7.4%). The total number of eyes affected and analysed was, therefore, 87.

**Fig. 1:**
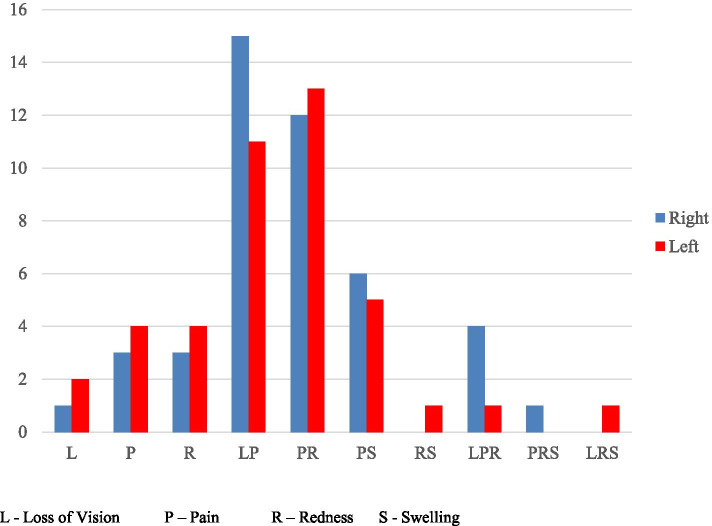
Distribution of Symptom Combinations in Affected Eyes

Pain was the commonest presenting complaint (74 eyes, 85%) and most patients had a combination of two presenting symptoms (63 eyes, 72%). The combination of pain with loss of vision had the highest frequency (26 eyes, 30%). The distribution of symptoms among the affected eyes is represented in Fig. 1.

The time interval between injury and presentation ranged from 2 h to 90 days with a median of 2 days (inter-quartile range 6.25 days). In all, 35 patients presented within 24 h, 27 patients presented later than 24 h but within a week and 19 patients presented after 1 week of injury (≤ 24 h = 43%; > 24 h but< 1 week = 34%; > 1 week = 23%). Of those presenting within 24 h of injury, 21 were male and 14 were female. Unpaired t-test was used for comparison of different groups and their approximate time of presentation after ocular trauma (see Table 2).

**Table 2:** Table of Statistical Significance

Groups	Test Value = 0					
	Mean	SD	t	*P*	95% CI	
					Lower	Upper
*Gender*						
Female (*N* = 35)	7.51	15.63	2.84	.008	2.14	12.88
Male (*N* = 45)	5.55	6.99	5.33	.000	3.45	7.65
*Educational Qualification*						
None (*N* = 4)	1.33	1.16	2.28	.107	−.53	3.18
Primary (*N* = 24)	6.06	6.97	4.26	.000	3.12	9.00
Secondary (*N* = 37)	4.99	5.82	5.21	.000	3.05	6.93
Tertiary (*N* = 13)	12.14	24.81	1.77	.103	−2.85	27.14
Postgraduate (*N* = 2)	9.63	13.26	1.03	.492	− 109.50	128.75
*First Aid*						
None (*N* = 48)	2.13	2.59	5.69	.000	1.37	2.88
Received (*N* = 32)	12.83	16.08	4.51	.000	7.03	18.63
*Form of First Aid*						
Hospital/Clinic (*N* = 13)	13.02	8.53	5.50	.000	7.86	18.18
Patent Medicine Dealer (*N* = 15)	13.82	22.38	2.40	.031	1.42	26.21
Self-Care/Home Care (*N* = 4)	8.50	3.87	4.39	.022	2.34	14.66
*Type of Injury*						
Open (*N* = 19)	3.28	5.64	2.54	.021	.56	6.00
Closed (*N* = 61)	7.38	12.73	4.53	.000	4.12	10.64

*N* Number, *SD* Standard deviation, *t* t-value, *P p* value, *CI* Confidence interval

Overall, males presented earlier with an average of 5.55 ± 6.99 days (*P* < 0.001). Inadditionthose with at least a secondary level as their highest educational qualification presented earlier than those with a primary level of education; 4.99 ± 5.82 days (*P* < 0.001).

Thirty (86%) of the patients that presented within the first 24 h of injury did not have any form of first aid/care prior to presentation. While 47 (76%) of those who presented within a week of injury did not seek first aid/ care prior to presentation. Generally, those who did not receive any form of first aid presented much earlier than those who did; 2.12 ± 2.59 days versus 12.83 ± 16.08 days (*P* < 0.001).

In all, 49 (60.5%) patients presented to the hospital without prior first aid/care. Of those that sought first aid/care prior to presentation, 15 (47%) received such care from a patent medicine dealer. The pattern of delayed presentation to an Ophthalmologist was similar for those who received first aid in a hospital or clinic by a non-specialist and those who consulted patent medicine dealers (*P* < 0.001).

**Fig. 2:**
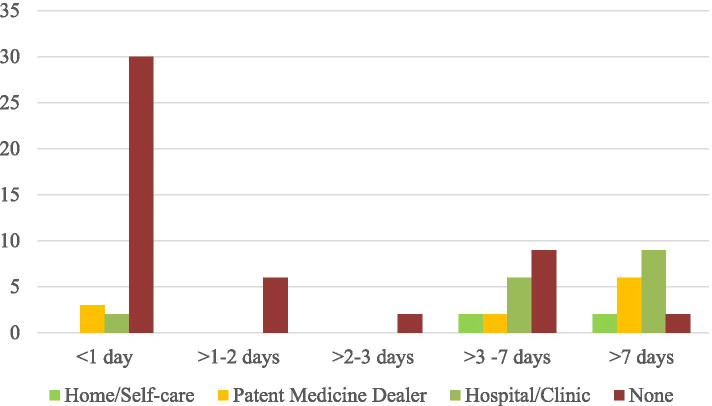
Pattern of Duration and Care Prior to Presentation

A greater proportion of open globe injuries presented within 24 h (76%) as compared to those with closed globe injuries (35%). Also a greater proportion of patients presenting with open globe injuries were male (15, 75%). The pattern of presentation and care seeking behavior is illustrated in Fig. 2.

The majority of injuries were closed globe injuries 66 (76%), occurred at home (35, 43%), resulted mainly from domestic accidents/ events (18, 22%), and were caused by blunt objects (46, 57%). Farming activities accounted for 7 of the 17 work-related injuries,thereby constituting the majority. The frequencies of these parameters are represented in Table 3.

**Table 3:** Place and Mechanism of Injury

	Number	Percentage
Place of Injury		
Farm	7	9%
Home	35	43%
Industry	6	7%
Road/Highway	17	21%
School	9	11%
Office	7	9%
Activity during Injury		
Assault/ Fight	16	19.8%
Domestic accident	18	22.3%
Play/ Recreation	15	18.5%
RTA-related	15	18.5%
Work Related	17	20.9%
Object Group		
Blunt	46	57%
Chemical	7	9%
Missile	10	12%
Sharp	16	20%
Thermal	2	2%
Type of Injury		
Closed	66	76%
Open	21	24%

The commonest domestic injuries were sustained from a fist or an open hand (26%). Other agents of domestic trauma included sticks/wood (23%), chemicals such as detergents (8%), burns (6%), stones (6%), bottle caps (6%), falls (6%), doors (6%) and pen/pencils (6%). Injuries from balls, metal bars and glass occurred least commonly. Contusion injuries on their own (48 eyes) or in combination with other forms of injuries (3 eyes), affecting a total of 51 (59%) eyes, constituted the majority of the type of injury identified. Penetrating injuries were more prevalent in the youngest age group. The age distribution of the type of injuries is shown in Table 4.

**Table 4:** Age Distribution of Ocular Injury (Birmingham Eye Trauma Terminology System)

	Age Groups			
	0–19 years	20–39 years	40–59 years	≥60 years
Contusion				
BETTS Class	17	20	12	2
Lamellar laceration	3	5	0	0
Penetrating	7	1	3	1
Perforating	0	3	0	0
Rupture	0	2	1	1
Superficial Foreign Body^a^	1	1	1	0
Intraocular Foreign Body	0	2	1	0
Chemical/Burns^a^	0	2	0	1
Total	28	36	18	5

^a^Not a component of BETTS classification

The most frequently occurring ocular findings were conjunctival (subconjunctival haemorrhage, 17; laceration, 3; conjunctivitis, 9) and corneal (ulcer, 10; laceration/perforation, 15). All ocular findings seen on assessment are detailed in Table 5.

**Table 5:** Ocular Findings in the affected eyes

Findings	Frequency (Eyes)
Lid	
Ecchymosis	10
Lid laceration/Abrasion	9
Conjunctiva	
Subconjunctival Haemorrhage	17
Traumatic Conjunctivitis	9
Conjunctiva Laceration	3
Superficial foreign body	3
Corneal	
Ulcer	10
Laceration	11
Perforation	4
Anterior Chamber	
Hyphaema	11
Uvea	
Traumatic Uveitis	12
Traumatic Mydriasis	6
Iris Tear	1
Choroidal Rupture	1
Uveal Prolapse	3
Lens	
Subluxated Lens	2
Dislocated Lens	1
Traumatic Cataract	1
Vitreous	
Vitreous Haemorrhage	1
Retina	
Traumatic Optic Neuropathy	2
Retinal oedema	2
Retinal Detachment	1
Globe	
Globe Rupture	4
Globe Perforation	3
Intraocular Foreign Body	3
Endophthalmitis	1
Orbit	
Orbital Fracture	1
**Total**^a^	132

^a^Some eyes had multiple findings

## Discussion

Ocular trauma remains a significant cause of monocular visual impairment worldwide [1] and in Nigeria [3, 4]. Tertiary hospitals provide specialist eye care services across the state at affordable rates and, between them, manage almost all eye trauma cases among the environs. The resultant incidence of 3.5% is similar to that obtained by Ajayi et al [24] in the South-West (3.8%) but significantly higher than that reported in the index study area in the eighties (1.16%) [22]. Increasing urbanization and motorization of the State over the years, with a corresponding increase in frequency of road traffic accidents and missile-related injuries, could account for this trend.

Most cases of ocular trauma were seen in the 10–19 years age group and over 75% of patients were under the age of 40 years. This is consistent with findings by Umeh [22] and Ezegwui [27]. Previous studies in other parts of Nigeria [24, 28–30] and Africa [31–33] also found adults of working age and children to be most affected. This age group has a higher tendency to be exposed to various agents of ocular injury at work or play. Unlike the study in Calabar [29], a bi-modal age distribution was not observed.

A male preponderance was observed in these patients, with a male to female ratio of 1.3:1. Similar findings have been reported in Europe [34, 35], Asia [36, 37], Africa [31–33] and previous studies in Nigeria [21, 22, 27, 28] This has been widely attributed to the risk-taking behaviour and tendency towards aggression of the male folk [21, 24]. In addition, men are more exposed to dangerous work environments and road traffic activities than women. The narrow margin, however, could be explained by the high proportion of domestic-related injuries which increasingly affects female individuals [15].

Several studies [27–29, 33] have found students, among other occupations, to have the highest predilection for ocular trauma. This was also the case in this study and can be attributed to their agility, lack of restraint and engagement in adventure-seeking activities. Similar to results by Megbelayin et al [29], civil service and trading were, also, recurring occupations among patients. This may be due to the demographic make-up of the State.

As found in past literature [21–24, 27–29, 38], majority of cases were unilateral; buttressing the finding of ocular trauma as a leading cause of monocular blindness. There was a slight right eye predominance in this study, as was also observed by Omolase et al. [21]Megbelayin et al [29] and Okoye et al [39] similarly reported more frequent right eye involvement. This may be attributed to a predominance of right-handedness among individuals when wielding tools, instruments or potential weapons. As a result, the same side of the eyes tends to be more frequently affected. The study by Okoye et al [40] on open globe injuries reported a predominance of left eye involvement and Ajayi et al [24] observed no significant difference in right-left distribution of the affected eye. Bilateral involvement was mainly as a result of chemical eye injury and gunshot pellets, as previously documented by Adepoju et al [19] and Bosaana et al. [33]

“Domestic activities” were the most common activity leading to ocular trauma and frequent causative agents were blunt objects. Most cases of ocular injury occurred in the home. These findings are supported by reports from Ezegwui [27], Abraham et al [20], Omolase et al [21] and Bonsaana et al. [33] Ajayi et al [24] in South western Nigeria and Alemayehu et al [32] in Addis Ababa, found work-related injuries to predominate,which is contrary to findings in this study. However, agriculture-related injuries made up the bulk of work-related injuries, as white collar jobs pose a minimal risk for eye injury. As found in the earlier mentioned studies [21, 27, 33], fight/assault and road traffic accident-related eye injuries were found to be on the increase. With increasing incidence of armed robbery, civilian-combats and highway constructions, this is not unexpected.

Most patients sustained closed globe injuries and contusions were the most recurring type of injury; in keeping with findings by other authors [22, 24, 27, 33]. This could possibly be explained by the fact that most agents of injury were blunt objects such as fists, wood and blunt objects of road traffic accidents. Most cases of corneal lacerations occurred in children, as supported by a study in Port Harcourt [30], during play with a sharp object.

Patients who presented within 24 h of the injury constituted 43% and most presented before 1 week. A similar trend was observed in Ethiopia [32] where 44% of patients presented within a day of the injury. Studies from Owo, Nigeria [21], also reported that most of the subjects presented within 24 h which accounted for the good visual outcome, eventually. In the previous prospective study in Enugu [22], most patients presented after 1 week of the injury. There has been notable improvement in the time of presentation over the last three decades. This remarkable positive change in health seeking behaviour among subjects may be due to increasing awareness and education of the city dwellers, as level of education has been significantly associated with the early presentation of some patients [28].

## Conclusion

This study has shown that there has, indeed, been a change in the pattern as well as the incidence of eye injuries in Enugu State. Though demographic distribution of age and gender have remained similar to that of past studies in the area, it has revealed a drop in farm-related injuries with assault and road traffic accidents emerging as important causes. In addition, there has been an improvement in the health seeking behaviour of people living in Enugu and this may reflect positively on the uptake of recommended preventive strategies such as mass education on possible causative agents and activities, implementation of mandatory protective eyewear in the workplace as well as legislations enforcing road traffic safety measures.

## Data Availability

The data for this research is available from the corresponding author.

## References

[1] Aghadoost D (2014). Ocular trauma: an overview. Arch Trauma Res.

[2] Négrel AD, Thylefors B (1998). The global impact of eye injuries. Ophthalmic Epidemiol.

[3] Azonobi IR (2010). Monocular blindness in Bayelsa state of Nigeria. Pan Afr Med J.

[4] Duke R, Lewallen S, Courtright P (2014). Estimated prevalence of monocular blindness and monocular severe visual impairment in children of Cross Rivers State, Nigeria. Niger J Ophthalmol.

[5] Addisu Z (2011). Pattern of ocular trauma seen in Grarbet Hospital, Butajira, Central Ethiopia. Ethiop J Heal Dev.

[6] Duke RE, Faal HB, Duke RE (2007). Uniocular blindness among children in the Gambia. Port Harcourt Med J.

[7] Kuhn F, Morris R, Witherspoon CD, Mester V (2004). The Birmingham eye trauma terminology system (BETT). J Fr Ophtalmol.

[8] Scott R (2015). The ocular trauma score. Community Eye Heal J.

[9] Shah M, Shah S, Agrawal R, Patel K. Validation of a modified Birmingham eye trauma terminology classification for mechanical eye injuries. Trauma. 2018;20(3):217–20.

[10] Okeigbemen V, Kayoma D (2013). Visual outcome of childhood ocular injuries in a tertiary hospital in Benin city. Asian J Med Sci.

[11] Rao L, Ninan A, Rao K (2010). Descriptive study on ocular survival, visual outcome and prognostic factors in open globe injuries. Indian J Ophthalmol.

[12] Ojabo CO, Malu KN, Adeniyi OS (2015). Open globe injuries in Nigerian children: epidemiological characteristics, etiological factors, and visual outcome. Middle East Afr J Ophthalmol.

[13] Scott R (2011). The injured eye. Philos Trans R Soc Lond Ser B Biol Sci.

[14] Kanski J, Bowling B (2011). Clinical Ophthalmology: A Systematic Approach.

[15] Nwosu SN (1995). Domestic ocular and adnexal injuries in Nigerians. West Afr J Med.

[16] Okoye OI, Umeh RE (2002). Eye health of industrial workers in southeastern Nigeria. West Afr J Med.

[17] Kyari F (2015). Challenges of agriculture-related eye injuries in Nigeria. Community Eye Heal J..

[18] Oluyemi F (2011). Epidemiology of penetrating eye injury in Ibadan: a 10-year hospital-based review. Middle East Afr J Ophthalmol.

[19] Adepoju FG, Monsudi KF, Adekoya BJ (2014). Bilateral blindness from ocular injury: a 15 year review. Afr J Trauma.

[20] Abraham EG, Ekanem US (2012). Prevalence of traumatic ocular injuries in a teaching hospital in south-South Nigeria – a 2 year review. Adv Trop Med Pub Heal Int.

[21] Omolase CO, Omolade EO, Ogunleye OT, Omolase BO, Ihemedu CO, Adeosun OA (2011). Pattern of ocular injury in Owo,Nigeria. J Ophthalmic Vis Res.

[22] Umeh RE (1988). Ocular trauma as seen in the University of Nigeria Teaching Hospital: a case study of eye injuries seen between January 1980 and May 1986 and a 1 1/3 year prospective study of cases seen between June 1986 and October 1987.

[23] Okpala NE, Umeh RE, Onwasigwe EN (2015). Eye injuries among primary school children in Enugu, Nigeria: rural vs urban. Ophthalmol Eye Dis.

[24] Ajayi IA, Ajite KO, Omotoye OJ, Adeseye AI (2014). Epidemiological survey of traumatic eye injury in a Southwestern Nigeria tertiary hospital. Pakistan J Ophthalmol Pak J Ophthalmol.

[25] Adelson JD, Bourne RRA, Briant PS, Flaxman SR, Taylor HRB, Jonas JB (2021). Causes of blindness and vision impairment in 2020 and trends over 30 years, and prevalence of avoidable blindness in relation to VISION 2020: the right to sight: an analysis for the global burden of disease study. Lancet Glob Heal.

[26] Patel D (2015). Eye injuries: improving our practice. Community Eye Heal J..

[27] Ezegwui IR (2004). Eye injuries at Abakaliki Nigeria. Int J Ophthalmol.

[28] Rafindadi AL, Pam VA, Chinda D, Mahmud-Ajeigbe FA (2013). Orbital and ocular trauma at Ahmadu Bello University teaching hospital, Shika-Zaria: a retrospective review. Ann Niger Med.

[29] Megbelayin EO, Nkanga DG, Ibanga A, Okonkwo SN (2016). Pattern and causes of ocular injuries in Calabar, Cross River State. Nigeria. J Trauma Care..

[30] Adio O, Nwachukwu H (2012). Pattern of paediatric corneal laceration injuries in the University of Port Harcourt teaching hospital, Rivers state, Nigeria. BMC Res Notes.

[31] Mehari ZA (2014). Pattern of childhood ocular morbidity in rural eye hospital. Central Ethiopia BMC Ophthalmol.

[32] Alemayehu WT, Shahin S. Epidemiology of ocular injuries in Addis Ababa Ethiopia. J Ophthalmol East Cent South Africa. 2014;18(1).

[33] Bonsaana G, Nyenze E, Ilako D, Wanye S (2015). Review of ocular trauma in tamale teaching hospital, tamale, Ghana. J Ophthalmol East Cent South Africa.

[34] Soylu M, Sizmaz S, Cayli S (2010). Eye injury (ocular trauma) in southern Turkey: epidemiology, ocular survival, and visual outcome. Int Ophthalmol.

[35] Cillino S, Casuccio A, Di Pace F, Pillitteri F, Cillino G (2008). A five-year retrospective study of the epidemiological characteristics and visual outcomes of patients hospitalized for ocular trauma in a Mediterranean area. BMC Ophthalmol.

[36] Dandona L (2000). Ocular trauma in an urban population in southern India: the Andhra Pradesh eye disease study. Clin Exp Ophthalmol.

[37] Krishnaiah S, Nirmalan PK, Shamanna BR, Srinivas M, Rao GN, Thomas R (2006). Ocular trauma in a rural population of southern India: the Andhra Pradesh eye disease study. Ophthalmology.

[38] Onakpoya OH, Adeoye A, Adeoti CO, Ajite K (2010). Epidemiology of ocular trauma among the elderly in a developing country. Ophthalmic Epidemiol.

[39] Okoye OI (2006). Eye injury requiring hospitalization in Enugu, Nigeria. A one-year survey. Niger J Surg Res.

[40] Okoye O, Maduka-Okafor F, Eze B (2007). Open globe injuries. Niger J Surg Sci.

